# Seroprevalence of African Swine Fever in Senegal, 2006

**DOI:** 10.3201/eid1701.100896

**Published:** 2011-01

**Authors:** Eric M.C. Etter, Ismaila Seck, Vladimir Grosbois, Ferran Jori, Esther Blanco, Laurence Vial, Ayayi J. Akakpo, Rianatou Bada-Alhambedji, Philippe Kone, Francois L. Roger

**Affiliations:** Author affiliations: Centre International de Recherche Agronomique pour le Developpement UR 22 Animal and Integrated Risk Management, Montpellier, France (E.M.C. Etter, V. Grosbois, F. Jori, F.L. Roger);; École Inter-États des Sciences et Médecine Vétérinaires, Dakar, Senegal (I. Seck, A.J. Akakpo, R. Bada-Alhambedji, P. Kone);; University of Pretoria Mammal Research Institute, Pretoria, South Africa (F. Jori);; Centre International de Recherche Agronomique pour le Developpement UMR CIRAD-INRA "Control of Animal Diseases," Montpellier (L. Vial);; Ministerio de Ciencia e Innovación, Madrid (E. Blanco); Instituto Nacional de Investigación y Tecnología Agraria y Alimentaria, Madrid (E. Blanco);; Kasetsart University Faculty of Veterinary Medicine, Bangkok, Thailand (F.L. Roger)

**Keywords:** African swine fever, viruses, Senegal, prevalence, research

## Abstract

In Senegal, during 2002–2007, 11 outbreaks of African swine fever (ASF) were reported to the World Organisation for Animal Health. Despite this, little was known of the epidemiology of ASF in the country. To determine the prevalence of ASF in Senegal in 2006, we tested serum specimens collected from a sample of pigs in the 3 main pig-farming regions for antibodies to ASF virus using an ELISA. Of 747 serum samples examined, 126 were positive for ASF, suggesting a prevalence of 16.9%. The estimated prevalences within each of the regions (Fatick, Kolda, and Ziguinchor) were 13.3%, 7.8%, and 22.1%, respectively, with statistical evidence to suggest that the prevalence in Ziguinchor was higher than in Fatick or Kolda. This regional difference is considered in relation to different farming systems and illegal trade with neighboring countries where the infection is endemic.

## Abstract 2

Prevalence ranged from 7.8% to 22.1%, depending on region.

African swine fever (ASF) is a disease caused by a DNA virus in the family *Asfarviridae*. The disease is highly contagious and often lethal for pigs and is of considerable economic importance, due to the direct loss of animals as well as resulting trade restrictions. No vaccine is available against the virus. The epidemiology of ASF is complex, transmission is direct and vector-borne, and the disease has well recognized sylvatic and domestic cycles.

ASF is currently considered enzootic in eastern and southern Africa, and the epidemiologic cycles of importance in many of the countries in these regions are well understood (*1*). In contrast, little is known about the epidemiology of the infection in West Africa, despite evidence of considerable spread of disease in this region in the late 1990s. Since it was first identified in Senegal in 1959, frequent reports of outbreaks of ASF in the country have been made to the World Organisation for Animal Health (OIE). Since 1986, a total of 54 outbreaks have been reported, with periods of frequent reports (19 outbreaks during 1986–1989; 15 outbreaks during 1999–2003) and periods with a lower frequency of reports (15 outbreaks during 1989–1998; 5 outbreaks during 2004–2006). The sylvatic cycle likely plays little role in the epidemiology of ASF in West Africa.

The suggestion has been made that in Senegal a domestic cycle of infection involving ticks may be possible because of the enzootic nature of disease in the country and the identification of infected soft ticks in some pig pens (*2*). Nevertheless, a pig-to-pig domestic cycle appears to be the main cycle of infection in the country, due to the large free-ranging pig population, along with regular reintroduction from disease-endemic countries. The pig sector plays a large part in the economy in several regions of Senegal and has dramatically increased in size in recent years (from 191,000 pigs in 1997 [*3*] to 320,000 in 2005 [*4*]). The consequences of ASF outbreaks in many countries are catastrophic, with major economic losses in developing countries, and considerable social effects may result: the loss of employment for farm workers, the loss of an major source of income for farmers, the loss of a major source of high quality and cheap protein for poor communities, and the consequences for traditional ceremonies (for which pigs are often required, as is seen in Cameroon and Côte d’Ivoire) (*5**,**6*).

Because of religious dietary restrictions, pigs within Senegal are principally clustered within regions containing the majority of the non-Muslim population, such as the population of the Casamance (the Ziguinchor and Kolda regions), and in areas where tourism has increased the demand for pork, such as Sine Saloum (the Fatick region). Although ASF has been identified as one of the 6 major diseases in need of epidemiologic surveillance in Senegal, few structured surveys have been conducted (*7*). A problem of underreporting of disease in the country was identified by Lefèvre in 1998; he explained that a large gap exsisted between declarations and reality (*3*). To date, no statistical data are available on the epidemiology of ASF in Senegal. Therefore, this study was designed to fill that information gap and document the seroprevalence of African swine fever in Senegal.

## Materials and Methods

### Sampling Protocol

The sampling protocol adopted in this study was based on the information obtained during a survey of pig production systems in Senegal (*7*). As mentioned earlier, the regions of Fatick, Kolda, and Ziguinchor were selected for the study because they corresponded to the area with the largest pig populations in Senegal. Kolda and Ziguinchor are located in the area of Casamance, which borders both Guinea-Bissau and the Gambia (within which, ASF is enzootic); and Fatick (an area where pig production is more dedicated to pig fattening) is located between Casamance and Dakar, the main area of pork meat consumption. Casamance and the Fatick region contain 75% of all pigs in Senegal (*4*) (Figure).

**Figure:**
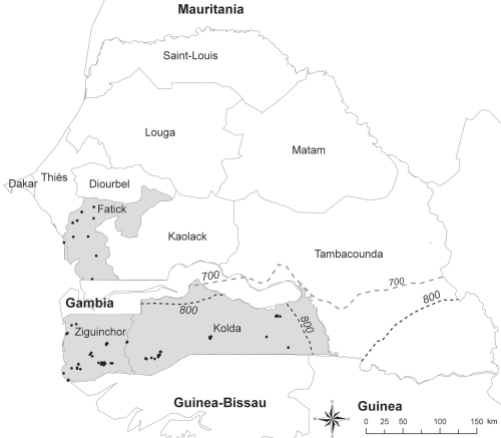
Sampled villages (black dots) in the 3 main regions of Senegal for pig production, Fatick, Ziguinchor, and Kolda (gray shading). Dashed lines indicate the 700 mm (gray) and 800 mm (black) rainfall isohyets for 2006. The southern limit range of *Ornithodoros sonrai* tick distribution (750 mm) can be estimated.

Free-range farming has been identified previously as the most widespread pig farming system in Senegal, with a recent study estimating that 76% (95% confidence interval 72%–80%) of all farms in the country were free-range systems (*7*). Therefore, villages as well as individual farms were considered as potential clusters.

A multistage sampling approach was adopted: the random selection of villages was followed by the random selection of farms within these villages. To estimate the required sample size, a prevalence of 50% was assumed (to maximize the required sample size), with a required precision of 6% and an α-error of 5%. Villages were considered as clusters of animals, and a decision was made to sample 10 pigs per village, from as many different farms as possible to maximize the representativeness of the sample. The formulas used in determining sample size, while accounting for clustering at the village level, are shown below (*8**,**9*):
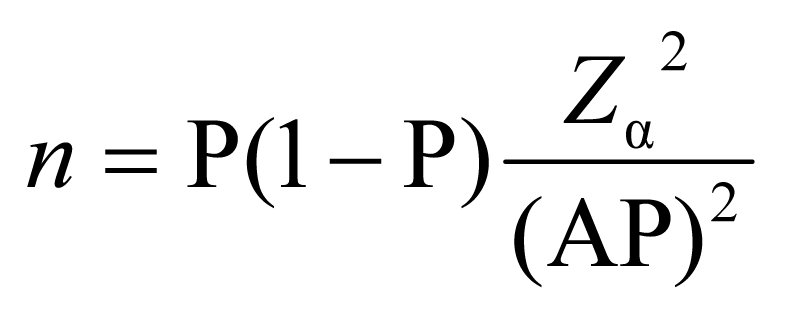

*n*′ = *n*(1 + ρ(*m* – 1))

where *n* is the sample size without correction, *Z*_α_ is the percentile point relating to the required α error under the *z* distribution, AP is the absolute precision, P is the estimated prevalence, *n*′ is the final sample size, ρ is the intracluster correlation, and *m* is the number of units sampled in each village. An intracluster correlation of 0.2 was used, because it rarely exceeds this value in the case of infectious disease (*10*).

The required sample size was 748, from a total of 75 villages; 756 pigs were actually sampled, from 82 villages and 205 farms (Table 1; Figure). Due to logistical and cost issues, the number of villages sampled in each region was not equal. Following a single exposure to ASF virus, antibodies will persist for at least 2 years (*11*). Therefore, to reduce the effect of past exposure to virus, only pigs from 6 months to 2 years of age were sampled. All pigs appeared healthy at the time of sampling.

**Table 1:** Numbers of villages and farms sampled and number of realized samples for African swine fever virus in each study region, Senegal, 2006

Characteristic	Fatick	Kolda	Ziquinchor
No. villages	15	24	43
No. farms	72	64	69
Realized samples	152	286	318

### Dates and Laboratory Analysis

Sampling was undertaken in May and July 2006, during the dry season. Blood samples were collected from the jugular vein in plain tubes and were centrifuged to obtain serum. Serologic analysis was performed by using an Ingezim PPA Compac 1.1.PPA K3 ELISA kit (Ingenasa, Madrid, Spain), which is a blocking ELISA that uses a purified protein extract from the virus (VP73) as the antigen. According to C. Gallardo (researcher at Centro de Inestigacion en Sanidad Animal, Madrid, Spain; pers. comm.) the sensitivity and specificity of this test were both in the region of 95% to 98%. The apparent prevalence estimates were therefore corrected to give the true prevalence by using the following formula (*9*):
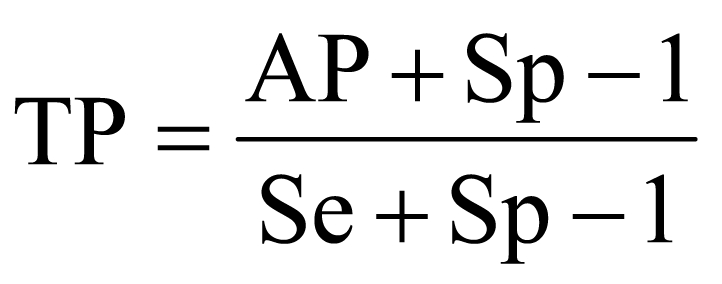
where TP is the true prevalence; Se and Sp are the sensitivity and specificity of the test, respectively; and AP is the apparent prevalence according to the test results.

### Statistical Analysis

To account for clustering within villages and farms when estimating the regional prevalence, a general linear mixed model method was adopted; the ‘lme4’ package within R software was used (www.r-project.org). Farms were nested within villages, and both were modeled as random effects. A z test was conducted to compare these random effects, to evaluate whether evidence of clustering of seropositivity could be found within villages and farms. The prevalence estimates in different regions were then compared by using the method described by Altman and Bland (*12*).

## Results

The z test found evidence of clustering of seropositivity within villages and farms (p<0.05). The general linear mixed model method gave seroprevalence estimates of 13.3%, 7.8%, and 22.1% for the regions of Fatick, Kolda, and Ziguinchor, respectively (Table 2). The prevalence estimate for Ziguinchor was significantly higher than that for Kolda (p<0.05) and that for Fatick (p<0.1).

**Table 2:** Individual prevalence of African swine fever in main pig breeding regions, Senegal, 2006

Region	No. pigs sampled	Estimated individual prevalence, %	95% confidence interval for the apparent prevalence	Uncertainty interval for the true prevalence, %
Fatick	149	13.3	8.0–21.2	3.2–20.6
Kolda	281	7.8	4.9–12.2	0–11.0
Ziguinchor	317	22.1	16.9–28.3	12.8–28.3

Test sensitivity and specificity were accounted for to estimate the true seroprevalence for each region. We gave the smallest and higher value taking into account the uncertainty around the true value of sensitivity and specificity. Following this process, the seroprevalence estimates for Fatick, Kolda, and Ziguinchor were expanded to range from 8.9% to 12.1%; from 3.0% to 6.2%; and from 18.3% to 21.6%, respectively. 95% confidence intervals for these estimates are shown in Table 2.

## Discussion

The results of this study are corroborated by unpublished data from the Senegalese Institute of Agricultural Research regarding the prevalence of ASF in the Ziguinchor region (*13*). Previous prevalence data from the Kolda region were not available. In 1988, no ASF antibodies were detected in pigs in the Fatick region; whereas 18 years later, the disease seemed to circulate periodically (*13*). This was probably due to virus spread from Guinea-Bissau (where the disease is enzootic) to Casamance, and then to the Gambia (where persons originally from Guinea Bissau, Casamance, and the Gambia all produce pigs).

Haresnape et al. conducted a seroprevalence study for ASF virus in Malawi and also collected information from pig owners about clinical signs and illness duration (*14**,**15*). The virus was considered enzootic in the western part of the central region between 1981 and 1986, where prolonged outbreaks of ASF were common. However, in the southern regions, ASF occurred in intermittent epizootics, and no evidence of ASF virus circulation was found in the northern region. On the basis of these findings, ASF virus strains of low virulence were believed to be present in the country (*14*), although no experimental proof was given. Infected meat introduced from affected areas was proposed to be the main source of ASF outbreaks, although warthogs tested positive for antibodies against ASF virus in the southern region. This finding suggested that a wildlife reservoir could play a role in the epidemiology of ASF in the country. In 9 of the 24 districts of Malawi, ASF virus was also detected in *Ornithodoros moubata* ticks, which are likely acting as a virus reservoir and vector of ASF virus (*16*).

The ASF situation in Mozambique was different from that in Malawi. In a study conducted in 1998, antibodies to ASF virus were detected in healthy pigs in the Angonia district, close to the Malawi border, indicating that these pigs survived an outbreak. However, experiments showed that this resistance was not highly heritable (*11*). ASF virus was considered enzootic in this district and was maintained in the population through a cycle involving domestic pigs only. No evidence of soft ticks, warthogs, or bush pigs was found in the area.

The current study has presented the estimated seroprevalence among pigs sampled within 3 regions and has accounted for clustering of seropositive individual pigs within farms and within villages. A more detailed characterization of the seroprevalence pattern could be conducted by estimating the presence of virus at different hierarchical aggregations; that is, the proportion of infected villages, the proportion of infected farms within infected villages, and the proportion of infected animals within infected farms. However, such an analysis, using hierarchical Bayesian modeling, for example, is beyond the scope of the present study (*17*).

True seroprevalence estimates were calculated by taking into account the sensitivity and specificity of the ELISA, which were estimated by using serum specimens from European domestic pigs (C. Gallardo, pers. comm.). Considering that ASF viruses currently circulating in West Africa are closely related to those circulating in Europe in the second half of the last century (*18*) (which are different from those currently circulating in Russia [*19*]), we can assume that the ELISA is appropriate for analyzing serum samples from Senegal.

*Ornithodoros sonrai* ticks containing remnants of ASF virus DNA have been identified in the Fatick region (*2*), which suggests the existence of an epidemiologic cycle in which ticks act as a reservoir, as occurs in eastern and southern Africa (*16*). However, the absence of a statistical association between the presence of ticks on pig farms and reported cases (or farmers’ suspicions of cases) of ASF, 3 years before the current survey, suggests that even if ticks were responsible for resurgence (*20*), they may not play a major role in the spread or emergence of ASF in this region, in contrast to the situation in Malawi (*16*). The Ziguinchor and Kolda regions are located below the 750 mm isohyet (≈13°30′N in Senegal; Figure), the southern limit of the reported geographic distribution of *O. sonrai* ticks (*21**,**22*). Therefore, the high prevalence of ASF in Ziguinchor is likely to be predominantly due to direct transmission between pigs, with little or no vector contribution. No bush pigs are present in Senegal, and according to local hunters and hunting settlements, warthogs in the Fatick region are scarce (with no available data on warthog numbers in the other regions). As such, the epidemiologic cycling of the virus in the country likely only involves domestic pigs, and the virus persists due to the large free-ranging pig population, as is the case in Mozambique (*11*). Further serologic studies involving warthogs are necessary to confirm the limited role of warthogs in the cycle of ASF in Senegal.

Although ASF virus can persist for long periods after infection and even recovery in pigs, seroprevalence estimates for the antibodies against the virus do not estimate the percentage of pigs with current infection, or even the percentage of carrier pigs. Rather, they indicate the percentage of pigs that have been exposed to the virus at some point in their lifetime. Bech-Nielsen et al. reported the detection of ASF virus in only 4.4% of carrier animals (*23*). Penrith et al. confirm also that fully recovered pigs apparently do not become long-term carriers (*11*). ASF antibody testing is recommended for the study of subacute and chronic forms of the disease (*24*). Also, the presence of antibodies against ASF virus does not imply that pigs are protected against new infections (*25*), since cellular immunity is essential for protection against ASF virus (*26*).

Considering that only pigs from 6 months to 2 years of age were tested, the pigs that tested positive must have become infected between 2004 and 2006. During this period, 5 outbreaks were declared in Senegal, with 646 cases and 561 deaths (*27**,**28*). When our data were compared with these official reports, we concluded that many cases were not declared by the farmers, possibly to avoid the costs of veterinary intervention and prohibition of animal movement.

Furthermore, our results suggest that these pigs survived virus infection, which contrasts with the widespread perception that mortality rates for ASF virus infection are high, approaching 80% (*25**,**29*). This high mortality rate mainly applies to the acute forms of the disease, which are more likely to be reported because of their dramatic effects on farms with large numbers of pigs or because they might have been responsible for the disappearance of farms with small number of pigs. Studies conducted in Spain and Portugal identified animals that have survived infections with ASF virus (*23*). Two possible explanations for the findings of the current study are that strains of ASF virus in Senegal have low virulence or that local breeds of pigs have some form of resistance to circulating ASF virus strains. In either case, the presence of healthy animals with antibodies suggests that ongoing circulation of ASF virus in the pig population in Senegal is a serious issue. This could explain the enzootic state of the disease in Senegal, even if stress factors are often needed to reactivate the transmission (*29*).

ASF virus strains of low virulence have been identified in various countries since 1984, and despite low virulence, could still maintain a high infectivity (*30*). In Senegal, however, in vivo tests on Large White pigs using ASF viruses isolated from pig leukocytes during 1987–1989 showed high virulence (*31*). These strains predominantly originated from Casamance (6 strains from 10 isolations), but more research, with experimental infection, is necessary to confirm whether new strains with low virulence are currently in Senegal. Indeed, outbreaks of ASF with high pig mortality rates have been reported in West Africa in the late 1990s: for example, in Côte d’Ivoire in 1996 (*5*), in Benin and Togo in 1997 (*3*), and in Nigeria during 1997–1998 (*32*). However, the 11 reported outbreaks of ASF in Senegal from 2002 through 2007 had mortality rates varying between 100% and 31% (*27**,**28*). Epidemiologic patterns of disease characterized by frequent outbreaks with low mortality have also been described in enzootic areas of southern Africa (Malawi [*14**,**33*] and Mozambique [*11*]).

Antibody-mediated resistance to ASF virus can be acquired through passive transfer of maternal antibodies or by previous infection with a virus of low pathogenicity or from low doses of highly virulent viruses (*11*). Development of protective immunity to ASF virus infection through either of these mechanisms could explain why healthy pigs with ASF antibodies were identified in the current study and why low mortality rates after exposure have been recorded in Senegal. These findings should be explored in further studies.

Significant differences in seroprevalence were observed between the 3 regions, with a higher seroprevalence identified in Ziguinchor than in Kolda or Fatick. The Ziguinchor region lies between the Gambia and Guinea-Bissau. ASF has been enzootic within Guinea-Bissau for years, and no efforts to control the disease have been reported (*3*). The legal and illegal trade of pigs between these neighboring countries could explain the higher prevalence observed in that region. Although the Kolda region also shares borders with these 2 countries, its eastern geographic location provides a drier and hotter climate, making it less favorable for pig farming. As a result of this and other economic issues, pig farming practices within the Kolda region are not as developed and organized as they are in the Ziguinchor region (*7*).

A study conducted in 1987 and 1988 found no evidence of seropositive animals among the 122 samples in the Fatick region (*13*). The current study, however, identified seropositive pigs in this region. This change may be a result of the development of pig trade between Fatick and the Ziguinchor region and the Gambia. Dakar, which lies north of the Fatick region, has one of the largest markets for pork in Senegal, and Fatick is therefore a crossing point for most of the pigs imported from the Gambia (*34*). Additionally, the recent development of the tourism industry in the Petite Côte and the Sine Saloum areas of the Fatick region over the past 10 years has increased the number of pigs in the region. The recent identification of ASF virus DNA in soft ticks in the Fatick region gives further evidence in support of spread of ASF virus into this region (*2*). The possible regular reintroduction of the virus from the Gambia or the Ziguinchor region could contribute to virus persistence in the region. Because trade is a likely factor affecting virus presence and persistence in all of the regions studied, further investigation of pig trade and the supply chains present in these regions is warranted.

Although ASF has been known to be enzootic in the Ziguinchor region for >10 years (*3**,**27*) (with all ASF reports to the OIE from 2002 through 2006 coming from this region), no cases have been reported in the Kolda region since 1996 (*27*). No cases from Fatick have been officially reported and reports from more northern regions (Thiès, Kaolack) have also been scarce. Lack of reporting of ASF cases could be explained by a limited interest by the authorities in the development of large-scale pig farms (*35*). A more accurate surveillance system, combined with compulsory reporting, could therefore help control the spread of the disease. Developing this system would require development of resources for the local veterinary services. A risk-based surveillance approach, involving the awareness of the pig farming community, would allow more efficient control of the disease, but will require further analysis of risk factors for infection in Senegal. A new public health policy regarding this issue, which includes a strategy of information dissemination about the disease and its risk factors among the pig farming community, is urgently needed.
